# Evaluation of Short-Term Effects on Colorectal Surgery Elective Patients after Implementing a Patient Blood Management Program: A Multicenter Retrospective Analysis

**DOI:** 10.3390/jcm13154447

**Published:** 2024-07-29

**Authors:** Filippo Carannante, Gabriella Teresa Capolupo, Manuel Barberio, Amedeo Altamura, Valentina Miacci, Martina Zenobia Scopigno, Erika Circhetta, Gianluca Costa, Marco Caricato, Massimo Giuseppe Viola

**Affiliations:** 1 Colorectal Surgery Clinical and Research Unit, Fondazione Policlinico Universitario Campus Bio-Medico, University Campus Bio-Medico, Via Àlvaro del Portillo 200, 00128 Rome, Italy; g.capolupo@policlinicocampus.it (G.T.C.); valentina.miacci@unicampus.it (V.M.); martina.scopigno@gmail.com (M.Z.S.); g.costa@policlinicocampus.it (G.C.); m.caricato@policlinicocampus.it (M.C.); 2 Research Unit of Surgery, Department of Medicine and Surgery, Università Campus Bio-Medico di Roma, Via Alvaro del Portillo, 21, 00128 Roma, Italy; 3 General Surgery Department, Ospedale Card. G. Panico, 73039 Tricase, Italy; manuel.barberio@ircad.fr (M.B.); amedeo.alta@gmail.com (A.A.); masgiuviola@yahoo.it (M.G.V.); 4 Haematology and Stem Cell Transplant Unit, Fondazione Policlinico Universitario Campus Bio-Medico, Via Àlvaro del Portillo 200, 00128 Rome, Italy; e.circhetta@policlinicocampus.it; 5 Department of Life Science, Health and Health Professions, Link Campus University, 00165 Roma, Italy

**Keywords:** colorectal surgery, patient blood management, transfusion, anemia, surgical complication

## Abstract

**Introduction**: Patients who undergo surgery may require a blood transfusion and patients undergoing major colorectal surgery are more prone to preoperative and perioperative anemia. Blood transfusions have, however, long been associated with inflammatory and oncological complications. We aim to investigate the effects of an optimal implementation of a patient blood management (PBM) program in our hospital. **Methods**: This study retrospectively reviewed data from two different prospectively maintained databases of all patients undergoing elective major colorectal surgery with either a laparoscopic, open, or robotic approach from January 2017 to December 2022 at two different high-volume colorectal surgery Italian centers: the Colorectal Surgery Unit of Fondazione Policlinico Campus Bio-Medico in Rome and the Colorectal Surgery Unit of Fondazione Cardinale Panico in Tricase (Lecce). Our study compares the first group, also known as pre-PBM (January 2017–December 2018) and the second group, known as post-PBM (January 2021–December 2022). **Results**: A total of 2495 patients, who satisfied the inclusion and exclusion criteria, were included in this study, with, respectively, 1197 patients in the pre-PBM group and 1298 in the post- PBM group. The surgical approach was similar amongst the two groups, while the operative time was longer in the pre-PBM group than in the post-PBM group (273.0 ± 87 vs. 215.0 ± 124 min; *p* < 0.001). There was no significant difference in preparatory Hb levels (*p* = 0.486), while anemia detection was significantly higher post-PBM (*p* = 0.007). However, the rate of transfusion was drastically reduced since the implementation of PBM, with *p* = 0.032 for preoperative, *p* = 0.025 for intraoperative, and *p* < 0.001 for postoperative. **Conclusions**: We confirmed the need to reduce blood transfusions and optimize transfusion procedures to improve short-term clinical outcomes of patients. The implementation of the PBM program was associated with a significant reduction in the rate of perioperative transfusions and an increase in only appropriate transfusions.

## 1. Introduction

Postoperative anemia is present in up to 90% of patients undergoing major surgery [1]. A significant portion of patients with colorectal cancer also have preoperative anemia, which can be attributed to tumor bleeding, a chronic pro-inflammatory state, malnutrition, or neoadjuvant therapy [2]. The effect of these factors can last for few weeks after major surgery and aggravate postoperative iron deficiency anemia (IDA). The immediate and most widely used treatment for postoperative anemia is packet red blood cell (PRBC) transfusion, which leads to the fastest but only transient correction of anemia and does not represent the etiological treatment of IDA. It is well known that blood transfusion is a common practice among hospitalized patients, especially amongst surgical patients who show a higher risk of developing perioperative anemia due to a pre-existing or acquired anemia, and it may prevent severe complications during uncontrolled bleeding. It is considered a life-saving treatment. 

However, a transfusion of packed red blood cells (PRBCs) is not without risks, and it has been known to increase risks in systemic inflammatory response, postoperative complications, and long-term outcomes in oncologic surgical patients. Even a single unit of transfused PRBCs has been shown significantly to increase 30-day mortality, composite mortality, pneumonia, and sepsis [3].

The association between anemia and an increased risk of blood transfusion has been confirmed by clinical studies in surgical oncology [4]. Anemia may also be associated with increased postoperative morbidity and mortality, which is based on a meta-analysis of oncology studies that included patients who underwent surgery for cancer. Preoperative anemia was associated with a 35 per cent increased risk of one major postoperative complication and a 42 per cent increased risk of death [5]. 

PBM is a multimodal approach based on the optimization of preoperative hemoglobin levels, the appropriate management of intraoperative blood loss, and correct postoperative anemia management. 

However, limited evidence on postoperative anemia management is currently available, indicating that, despite its high prevalence with a negative impact on clinical and long-term outcomes, more attention needs to be given to this topic. Therefore, a postoperative anemia management protocol is strongly needed to minimize its impact on clinical outcomes and to allow a faster recovery.

Patient blood management (PBM) is an evidence-based multimodal approach devoted to blood transfusion optimization. It was introduced in the last decade, starting in North America, to reduce the use of blood products and improve tolerance to anemia; PBM for surgical patients is based on three fundamental pillars according to the World Health Organization (WHO): the optimization of the preoperative red cell mass, the minimization of blood loss, and the correct management of postoperative anemia [6].

A PBM program was introduced in our institute to reduce unnecessary blood transfusions and optimize major surgical procedures. PBM for surgical patients is based on three factors: the optimization of the preoperative red cell mass, minimizing blood loss, and the correct management of postoperative anemia. These are the three pillars of patient blood management according to the WHO [6]. 

According to different studies, establishing a PBM program can reduce mortality up to 68%, reoperation up to 43%, readmission up to 43%, composite morbidity up to 41%, infection rate up to 80%, the average length of stay by 16–33%, transfusion from 10% to 95%, and costs from 10% to 84% after major surgery [7].

This study offers an analysis of the PBM program’s implementation and the pertinence of PRBC transfusions on postoperative outcomes in the short term of patients who underwent major colorectal surgery in two high-volume colorectal surgery Italian centers in Rome and Tricase, respectively. We aim to investigate the effects of implementing a PBM program on our blood transfusion practice and on short-term postoperative outcomes.

## 2. Materials and Methods

We performed a retrospective analysis of two different prospectively maintained databases of all patients undergoing elective major colorectal surgery with either a laparoscopic, open, or robotic approach from January 2017 to December 2022 at two different high-volume colorectal surgery Italian centers: the Colorectal Surgery Unit of Fondazione Policlinico Campus Bio-Medico in Rome and the General Surgery Department of Fondazione Cardinale Panico in Tricase (Lecce).

This study was approved by the institutional review board of Fondazione Cardinale Panico.

Both institutions improved their PBM system in January 2019. A multidisciplinary working group was established to promote the most appropriate transfusion practices in accordance with PBM approaches. The protocol was based on the most important items of PBM [8,9] and the blood transfusion policy was defined according to the specific indications of current transfusion guidelines for red blood cells, platelets, and plasma.

These indications are preoperative, intraoperative, and postoperative to prepare the patient for the surgical procedure and to prevent and manage potential blood losses.

In particular, preoperative indications include the identification of patients with anemia, at least 15 days before surgery, referring patients to the Transfusion Medicine Department in order to identify iron and folic acid vitamin B12 deficiencies and to create patient-specific plans to minimize blood loss; intraoperative indications include maintaining careful hemostasis and meticulous surgery, including the use of hemostatic agents and promoting the routine use of minimally invasive surgeries; and postoperative indications include monitoring for postoperative bleeding and preventing infections and avoiding iron deficiency.

The patients were categorized into two groups: the first included patients who underwent surgery between January 2017 and December 2018, before the implementation of the PBM program, and the second included patients who underwent surgery from January 2021 to December 2022, after the implementation of the PBM program. They are called pre-PBM and post-BPM. The factors that were considered in this study were the following: the socio-demographic profile, the anamnesis, the American Society of Anesthesiologists (ASA) score, neo-adjuvant chemotherapy, tumor location or the inflammatory disease extent, the staging of the tumor, the surgical approach, the length of the operation, the estimated blood loss, Hb levels prior to the surgery and the number of PRBCs transfusions. The study inclusion criteria were as follows: (1) colorectal resection, (2) elective surgery, (3) histological evidence of adenocarcinoma, (4) transanal approach, and (5) a history of inflammatory bowel disease (6). The exclusion criteria were (1) emergency surgery, (2) transanal minimally invasive surgery (TAMIS) or transanal excision (TAE), (3) the combined resection of other major organs (i.e., lungs or liver), (4) a history of bone marrow-related disease, and (5) a history of chronic renal failure. To appropriately study the rate of transfusions, the Hb levels and the number of PRBC units administered when transfusing each patient were studied.

Postoperative outcomes were analyzed to compare short-term postoperative outcomes between the two groups.

## 3. Statistical Analysis

Patients’ characteristics were summarized using basic descriptive statistics. Continuous variables were presented as mean ± standard deviation values and compared using a *t*-test on individual samples. For categorical data, the χ^2^ test was used, and the results were expressed as percentages.

Statistical analysis was carried out using StataCorp2019 STATA Statistical Software: release 16 (College Station, TX, USA: StataCorp LLC). All tests were considered statistically significant with a *p* value ≤ 0.05.

## 4. Results

A total of 2495 patients, who satisfied the inclusion and exclusion criteria, were included in this study, with, respectively, 1197 patients in the pre-PBM group and 1298 in the post-PBM group. Preoperative and intraoperative patient characteristics are reported in Table 1. There was no significance in age difference (*p* = 0.267) or gender (*p* = 0.431). The post-PBM group showed an overall higher ASA score (*p* = 0.004).

The surgical approach was also similar amongst the two groups, where the laparoscopic approach was used in 81.7% and 80.7% of the patients, respectively. The robotic approach was used in 13.9% and 16.7% of the patients, respectively, while the open approach was used in a small minority of the patients. The operative time was longer in the pre-PBM group than in the post-PBM group (273.0 ± 87 vs. 215.0 ± 124 min; *p* < 0.001).

There was no significant difference in preoperative Hb levels (*p* = 0.486), while anemia detection was significantly higher post BPM (*p* = 0.007). However, the rate of transfusion was drastically reduced since the implementation of the PBM program, with *p* = 0.032 for pre-operative, *p* = 0.025 for intraoperative and *p* < 0.001 for postoperative.

Moreover, the total number of transfused PRBC units was reduced from 944 U before PBM to 252 U after the implementation of the PBM program (Table 2).

Lastly, the postoperative complications did not show any statistically relevant differences between the pre-PBM and post-PBM groups (29.8 vs. 27.6%; *p* = 0.412). However, reductions in anastomotic leakage (7.3 vs. 6.8%; *p* = 0.08) and length of hospital stay after surgery (LOS) (10.2 ± 7.1 vs. 8.2 ± 5.7 days; *p* = 0.09) were detected but not statistically significant. There was no significant difference in any of the other complications or their grading according to the Clavien–Dindo system between the two groups.

The same was seen for the rate of re-intervention, re-admission to hospital and mortality, all at 30 days, between the two groups (Table 3).

## 5. Discussion

Our study confirmed the positive outcome of the PBM program in the optimization of the perioperative transfusion rate in patients undergoing major colorectal surgery.

The implementation of the PBM program was associated with a significant reduction in the rate of perioperative transfusions and an increase in only appropriate transfusions. Optimizing transfusion rates is important for the care of patients requiring essential transfusions and for reducing medical costs.

Previous studies have confirmed that the introduction of PBM can reduce the number of unnecessary blood transfusions. Gani et al. [10]. retrospectively evaluated the results of PBM implementation in an ample pool of surgical patients (*n* = 17.114) and confirmed an increase in the number of “appropriate” hemoglobin transfusions (Hb trigger: <7 g/dL; *p* < 0.001) and a decrease in the rate of inappropriate hemoglobin transfusions (Hb trigger: ≥8.0 g/dL; *p* < 0.001). The authors reported a reduction in the rate of transfused patients with Hb ≥ 9 or 10; *p* < 0.001. These results clearly show the positive role of PBM on the optimization of transfusional procedures based on the evidence, with possible repercussions on short-term postoperative outcomes.

In a retrospective study on patients who had undergone major colorectal surgery for colorectal cancer, McSorely et al. [11] reported that perioperative blood transfusions were associated with an increased systemic postoperative inflammatory response (measured with c-reactive protein), postoperative complications (*p* = 0.0197), anastomotic dehiscence (*p* = 0.021), and hospital stay (*p* = 0.011), without any change in the rate of long-term survival (overall survival or cancer-specific survival) in the compared groups.

These results confirm the need to reduce blood transfusions and optimize transfusion procedures to improve short-term clinical outcomes of patients. Multiple studies have confirmed an association between blood transfusions and infective postoperative complications, anastomotic leakage, and recurrence or survival [12,13,14,15]. Pang et al. also found a correlation between the overall survival and volume of transfused blood [4]. However, Hanna et al. [16] reported that blood transfusion was associated with a reduction in disease-free survival, but a decreased overall survival and an increase in hospital stay was associated with curative rectal resection due to cancer

Shin et al. [17] investigated the effects of PBM implementation in blood transfusion practice and on short-term postoperative outcomes and they concluded that the implementation of a PBM program was associated with a decrease in the total transfusion rate, a decrease in the Hb threshold before transfusion (Hb trigger), and an increase in the optimal transfusion rate, reducing postoperative LOS and anastomosis leakage.

In our study, PBM implementation was associated with a reduction in anastomotic leakage and LOS, with no statistical significance.

These results confirm the importance of reducing the number of blood transfusions and optimizing related surgeries to improve postoperative short-term outcomes.

A long-term follow-up study is needed to underline possible relationships between blood transfusions and long-term oncologic outcomes.

The relationship between blood transfusions and onco-infectious outcomes is yet to be determined. It has been hypothesized that an allogenic blood transfusion can compromise the adaptive immune response of the host, influencing the patient’s response to pathogens and other circulating or micrometastatic cancer cells [18].

Ercolani et al. [19] studied the effect of the introduction of PBM in abdominal surgery. They found a significant reduction in the number of patients receiving blood transfusions, with a strong tendency to minimize the use of blood products for most types of oncologic surgery.

When evaluating the current study, it should also be stated that before the implementation of the PBM program, both colorectal surgery departments were trying to reduce unnecessary blood transfusions to a minimum. Transfusion procedures can be further improved. It is expected that the current annual transfusion rate will be further decreased thanks to the continued improvement of the quality of the PBM program.

In the last 20 years, minimally invasive surgery has increasingly been used. Both laparoscopic and robotic surgery have demonstrated several advantages over “open” surgery, and, among these, the reduction in intraoperative blood loss appears consistent [20]. For these reasons, the use of a minimally invasive approach is also recommended in the PBM protocol in order to reduce the use of transfusions.

In recent years, the Enhanced Recovery After Surgery (ERAS) program has highlighted the impact of developing and implementing evidence-based best patient care pathways. Several studies have strongly demonstrated that preoperative anemia is independently associated with higher morbidity, mortality, and allogeneic blood transfusion rates. For these reasons, correcting preoperative anemia has been advocated by ERAS guidelines [21,22], national and worldwide healthcare programs, and many national professional societies [23,24].

Multimodal prehabilitation, which includes physical, nutritional, and psychological optimization, aims to strengthen physiological reserves by improving preoperative functional capacity. The ultimate goals are to better withstand surgical stress, reduce postoperative complications, and accelerate surgical recovery [25]. Within this clinical framework, many multimodal prehabilitation programs incorporate strategies to correct preoperative anemia, intending to enhance functional capacity and increase hemoglobin concentrations before surgery. Consequently, the ERAS program frequently recommends multimodal prehabilitation as a preoperative measure to better prepare patients for surgery and to minimize postoperative complications following abdominal surgery. To the best of our knowledge, no studies have been conducted to date to determine whether prehabilitation can improve anemia tolerance in surgical patients undergoing major abdominal surgery.

Moreover, trials investigating whether correcting preoperative anemia improves postoperative outcomes show contrasting results [26,27,28]. This might be due to the lack of PBM programs aiming at optimizing anemia and preventing excessive blood loss throughout the entire perioperative period rather than focusing solely on the preoperative period. In fact, several reviews and expert opinions suggest that PBM programs should be integrated within ERAS pathways to ensure that anemia and transfusion management are adequately addressed during the preoperative, intraoperative, and postoperative periods [29]. However, clinical trials evaluating the integration and impact of PBM programs in the context of the ERAS program are currently lacking. PBM programs are multimodal, patient-centered pathways with an interdisciplinary approach for patients undergoing major surgery. PBM is effective in reducing perioperative complication rates, maintaining patients’ own blood mass, thereby improving clinical outcomes and reducing costs [30]. Despite all these proven benefits, there are many knowledge gaps about PBM programs, particularly regarding whether integrating PBM as an element of the ERAS program can further enhance the benefits of ERAS pathways and at the same time facilitate the adoption of PBM programs [28]. The ERAS program has been one of the most recent significant innovations with a relevant impact on surgical practice. The ERAS program provides an evidence-based multimodal, multidisciplinary approach to attenuate perioperative stress and organ dysfunction and decrease the rate of postoperative complications, thereby enhancing recovery after surgery [31]. Considering the existing lack of evidence supporting the integration of PBM with the ERAS program, notwithstanding its strong physiological rationale, if integrating PBM with the ERAS program is proven beneficial, adequate institutional resources should be allocated to successfully implement clinical practice changes. Finally, the implementation of PBM within the ERAS program might be a further opportunity to facilitate the uptake of fast track pathways, redesign perioperative settings with evidence-based interventions, and reduce unwanted clinical practice variability.

This study has several limitations. First, it is a retrospective analysis of a prospective database, and it is therefore possible that there is a certain degree of bias in the selection. Moreover, since this study has considered two separate but consecutive periods, the surgical techniques and management of the two groups could be skewed.

It is known that minimally invasive surgery is associated with lower perioperative blood loss and a reduced inflammatory response [32,33]. This could also have influenced the results of the current study, underestimating the possible effects of a blood transfusion on short-term postoperative outcomes; this aspect should be considered in future studies. However, our study also has several strengths. First, this is one of the largest cohorts that has been used to evaluate PBM in colorectal surgery. Secondly, it is probably the only the multicentric study on PBM. Thirdly, most patients underwent minimally invasive colorectal surgery, which is known to be associated with less blood loss but could also underestimate the possible effect on postoperative outcomes.

## 6. Conclusions

The following study demonstrates how, for patients undergoing major colorectal surgery, the implementation of PBM is associated with a reduction in the rate of total transfusion, a reduction in the minimum threshold for pre-transfusion Hb and an increase in the rate of optimal transfusions. The implementation of a PBM program is therefore demonstrated to be necessary to guarantee the correct use of preoperative transfusions, with relevant clinical and economic outcomes.

## Figures and Tables

**Table 1:** Preoperative and intraoperative patient characteristics. *p* value ≤ 0.05 were considered statistically significant.

Variable	Pre-PBM 1197 pts	Post-PBM 1298 pts	*p*
Age, years	67.8 ± 14.26	69.4 ± 13.0	0.267
Gender			0.431
Male	618 (51.6)	724 (55.7)	
Female	579 (48.3)	574 (44.3)	
BMI, kg/m^2^	25.84 ± 4.25	25.64 ± 4.66	0.669
ASA			0.004
1–2	777 (64.9)	646 (49.7)	
3–4	420 (35.1)	652 (50.2)	
Length of operation, min	273 ± 87	215 ± 124	<0.001
Tumor location			0.68
Right	468 (39.1)	499 (38.5)	
Left	461 (38.5)	483 (37.2)	
Rectum	268 (22.4)	316 (24.3)	
Surgical approach			0.26
Open	53 (4.4)	33 (2.6)	
Laparoscopic	978 (81.7)	1047 (80.7)	
Robotic	166 (13.9)	218 (16.7)	

**Table 2:** Preoperative anemia and the rate of perioperative transfusions. *p* value ≤ 0.05 were considered statistically significant.

Variable	Pre-PBM 1197 pts	Post-PBM 1298 pts	*p*
Preoperative Hb, g/dL	11.9 ± 2.2	12.1 ± 2.2	0.486
Anemia (%)	333 (27.8)	536 (41.3)	0.007
Transfusions, *n* (%)			
Preoperative	106 (8.9)	28 (2.1)	0.032
Intraoperative	159 (13.3)	57 (4.4)	0.025
Postoperative	262 (21.9)	87 (6.28)	<0.001
Total units	944	252	<0.001
Units per patient	2.7 ± 2.2	2.5 ± 2.1	0.43

**Table 3:** Postoperative characteristics.

Variable	Pre-PBM 1197 pts	Post-PBM 1298 pts	*p*
Overall complications (%)	357 (29.8)	359 (27.6)	0.64
Anastomotic leakage (%)	87 (7.3)	88 (6.8)	0.08
Postoperative bleeding (%)	20 (1.7)	19 (1.6)	0.71
Pneumonia (%)	18 (1.5)	14 (1.1)	0.93
Urinary tract infection (%)	17 (1.4)	15 (1.2)	0.85
Surgical Site Infection (%)	33 (2.8)	37 (2.9)	0.73
Clavien–Dindo grade			0.43
I	112 (31.4)	123 (34.4)	
II	138 (38.6)	134 (37.4)	
III	99 (27.8)	94 (26.4)	
IV	6 (1.8)	5 (1.6)	
V	3 (0.9)	1 (0.2)	
Hospital stay, days	10.2 ± 7.1	8.2 ± 5.7	0.09
30 days mortality, *n* (%)	16 (1.3)	11 (0.85)	0.65
30 days re-intervention, *n* (%)	127 (10.6)	166 (12.7)	0.52
30 days re-admission, *n* (%)	127 (10.6)	121 (9.3)	0.69

## Data Availability

The data presented in this study are available on request from the corresponding author.

## References

[1] Muñoz M., Acheson A.G., Auerbach M. (2017). International consensus statement on the peri-operative management of anaemia and iron deficiency. Anaesthesia.

[2] Knight K., Wade S., Balducci L. (2004). Prevalence and outcomes of anemia in cancer: A systematic review of the literature. Am. J. Med..

[3] Bernard A.C., Davenport D.L., Chang P.K., Vaughan T.B., Zwischenberger J.B. (2009). Intraoperative transfusion of 1 U to 2 U packed red blood cells is associated with increased 30-day mortality, surgical-site infection, pneumonia, and sepsis in general surgery patients. J. Am. Coll. Surg..

[4] Weber R.S., Jabbour N., Martin R.C. (2008). Anemia and transfusions in patients undergoing surgery for cancer. Ann. Surg. Oncol..

[5] Musallam K.M., Tamim H.M., Richards T., Spahn D.R., Rosendaal F.R., Habbal A., Khreiss M., Dahdaleh F.S., Khavandi K., Sfeir P.M. (2011). Preoperative anaemia and postoperative outcomes in non-cardiac surgery: A retrospective cohort study. Lancet.

[6] World Health Organization (2021). The Urgent Need to Implement Patient Blood Management: Policy Brief. https://www.who.int/publications/i/item/9789240035744.

[7] Farmer S.L., Trentino K.M., Hofmann A. (2015). A programmatic approach to patient blood management—Reducing transfusions and improving patient outcomes. Open Anesthesiol. J..

[8] Clevenger B., Mallett S.V., Klein A.A., Richards T. (2015). Patient blood management to reduce surgical risk. Br. J. Surg..

[9] Butcher A., Richards T. (2018). Cornerstones of patient blood management in surgery. Transfus. Med..

[10] Gani F., Cerullo M., Ejaz A., Gupta P.B., Demario V.M., Johnston F.M., Frank S.M., Pawlik T.M. (2019). Implementation of a Blood Management Program at a Tertiary Care Hospital: Effect on Transfusion Practices and Clinical Outcomes Among Patients Undergoing Surgery. Ann. Surg..

[11] McSorley S.T., Tham A., Dolan R.D., Steele C.W., Ramsingh J., Roxburgh C., Horgan P.G., McMillan D.C. (2020). Perioperative Blood Transfusion is Associated with Postoperative Systemic Inflammatory Response and Poorer Outcomes Following Surgery for Colorectal Cancer. Ann. Surg. Oncol..

[12] Amato A., Pescatori M. (2006). Perioperative blood transfusions for the recurrence of colorectal cancer. Cochrane Database Syst. Rev..

[13] Alzahrani A.A., Alturkistani S.A., Alturki H., Baeisa R.S., Banoun J.A., Alghamdi R.A., Alghamdi J.A. (2024). Evaluation of Factors That Contribute to Intraoperative and Postoperative Complications Following Colorectal Cancer Surgeries at King Abdulaziz University Hospital, Jeddah, Saudi Arabia. Cureus.

[14] Rohde J.M., Dimcheff D.E., Blumberg N., Saint S., Langa K.M., Kuhn L., Hickner A., Rogers M.A. (2014). Health care-associated infection after red blood cell transfusion: A systematic review and meta-analysis. JAMA.

[15] McDermott F.D., Heeney A., Kelly M.E., Steele R.J., Carlson G.L., Winter D.C. (2015). Systematic review of preoperative, intraoperative and postoperative risk factors for colorectal anastomotic leaks. Br. J. Surg..

[16] Hanna D.N., Gamboa A.C., Balch G.C., Regenbogen S.E., Holder-Murray J., Abdel-Misih S.R.Z., Silviera M.L., Feng M.P., Stewart T.G., Wang L. (2021). Perioperative Blood Transfusions Are Associated With Worse Overall Survival But Not Disease-Free Survival After Curative Rectal Cancer Resection: A Propensity Score-Matched Analysis. Dis. Colon. Rectum.

[17] Shin S.H., Piozzi G.N., Kwak J.M., Baek S.J., Kim J., Kim S.H. (2022). Effect of a Patient Blood Management system on perioperative transfusion practice and short-term outcomes of colorectal cancer surgery. Blood Transfus..

[18] Blumberg N., Heal J.M. (1994). Effects of transfusion on immune function. Cancer recurrence and infection. Arch. Pathol. Lab. Med..

[19] Ercolani G., Solaini L., D’Acapito F., Isopi C., Pacilio C.A., Moretti C., Agostini V., Cucchetti A. (2023). Implementation of a patient blood management in an Italian City Hospital: Is it effective in reducing the use of red blood cells?. Updates Surg..

[20] Solaini L., Bocchino A., Avanzolini A., Annunziata D., Cavaliere D. (2022). Ercolani G Robotic versus laparoscopic left colectomy: A systematic review and meta-analysis. Int. J. Colorectal Dis..

[21] Gustafsson U.O., Scott M.J., Hubner M. (2019). Guidelines for perioperative care in elective colorectal surgery: Enhanced recovery after surgery (ERAS^®^) society recommendations: 2018. World J. Surg..

[22] Ficari F., Borghi F., Catarci M. (2019). Enhanced recovery pathways in colorectal surgery: A consensus paper by the Associazione Chirurghi Ospedalieri Italiani (ACOI) and the PeriOperative Italian Society (POIS). G. Chir..

[23] National Institute for Health and Care Excellence Blood Transfusion. NICE Guideline. 18 November 2015. https://www.nice.org.uk/guidance/ng24/resources/blood-transfusion-pdf-1837331897029.

[24] American Society of Anesthesiologists Task Force on Perioperative Blood Management (2015). Practice guidelines for perioperative blood management: An updated report by the American society of anesthesiologists task force on perioperative blood management*. Anesthesiology.

[25] Scheede-Bergdahl C., Minnella E.M., Carli F. (2019). Multimodal prehabilitation: Addressing the why, when, what, how, who and where next?. Anaesthesia.

[26] Froessler B., Palm P., Weber I. (2016). The important role for intravenous iron in perioperative patient blood management in major abdominal surgery: A randomized controlled trial. Ann. Surg..

[27] Richards T., Baikady R.R., Clevenger B. (2020). Preoperative intra- venous iron to treat anaemia before major abdominal surgery (PREVENTT): A randomised, double- blind, controlled trial. Lancet.

[28] Talboom K., Borstlap W.A.A., Roodbeen S.X., FIT collaborative group (2023). Ferric carboxymaltose infusion versus oral iron supplementa- tion for preoperative iron deficiency anaemia in patients with colorectal cancer (FIT): A multicentre, open-label, randomised, controlled trial. Lancet Haematol..

[29] Guinn N.R., Goobie S.M. (2022). Patient blood management: The forgotten element of enhanced recovery after surgery programs. Anesth. Analg..

[30] Spahn D.R., Muñoz M., Klein A.A. (2020). Patient blood management: Effectiveness and future potential. Anesthesiology.

[31] Ljungqvist O., Scott M., Fearon K.C. (2017). Enhanced recovery after surgery: A review. JAMA Surg..

[32] Janež J., Korać T., Kodre A.R., Jelenc F., Ihan A. (2015). Laparoscopically assisted colorectal surgery provides better short-term clinical and inflammatory outcomes compared to open colorectal surgery. Arch. Med. Sci..

[33] Song X.J., Liu Z.L., Zeng R., Ye W., Liu C.W. (2019). A meta-analysis of laparoscopic surgery versus conventional open surgery in the treatment of colorectal cancer. Medicine.

